# Discordant lymphoma consisting of mediastinal large B-cell lymphoma and nodular sclerosis Hodgkin lymphoma in the right supraclavicular lymph nodes: a case report

**DOI:** 10.1186/s13000-015-0450-6

**Published:** 2015-12-29

**Authors:** Chun Zhang, Yuanxue Yi, Chunyan Chen, Jianrong Wang, Zhu Liu

**Affiliations:** Department of Pathology, Chongqing Corps Hospital of Chinese People’s Armed Polices, No. 90 Wei-guo Road, Nan’an, Chongqing, China; Department of Orthopaedics, Chongqing Corps Hospital of Chinese People’s Armed Polices, No. 90 Wei-guo Road, Nan’an, Chongqing, China

**Keywords:** Discordant lymphoma, Mediastinum, Large B-cell lymphoma, Supraclavicular lymph nodes, Nodular sclerosis Hodgkin lymphoma

## Abstract

**Background:**

Discordant lymphoma is defined by the simultaneous presence of two or more distinct types of lymphomas at different anatomic sites. With fewer than 20 studies reporting cases of discordant lymphoma to date, the incidence of this condition is believed to be very low.

**Case Presentation:**

Here, we report a case of discordant lymphoma in a 34-year-old female patient that involved mediastinal large B-cell lymphoma and nodular sclerosis Hodgkin lymphoma in the right supraclavicular lymph nodes. The patient presented with a mass in the mediastinum and enlargement of the right supraclavicular lymph nodes, but no obvious signs of lymphoma. Histological examination revealed that the encapsulated mediastinal mass contained medium- or large-size tumor cells with lightly stained cytoplasm and round vesicular nuclei as well as a high percentage of mitotic cells; strongly positive immunohistochemical staining for PAX5, CD20, and CD79a also was observed. Examination of biopsied right supraclavicular lymph node tissues revealed separation by collagen fibers, extensive inflammatory cell infiltration, and large-size tumor cells, such as Reed–Sternberg cells. These tissues stained strongly positive for PAX5 and CD30, weakly positive for CD15, and negative for Epstein-Barr viral RNA. We also found monoclonal gene rearrangement in the immunoglobulin heavy chain gene in the mediastinal large B-cell lymphoma, but no monoclonal gene rearrangement in the nodular sclerosis Hodgkin lymphoma. These findings suggested that these two lymphomas were not of a common clonal origin. The patient was treated by surgical excision of the mediastinal mass followed by radio-chemotherapy, and no metastasis or recurrence occurred during a follow-up period of 32 months.

**Conclusion:**

A review of previously reported cases indicated that the clinical manifestations and pathological features of discordant lymphoma are diverse due to variation in the types of lymphomas involved. Physicians must have an awareness of discordant lymphoma to avoid incorrect and missed diagnoses, especially considering that the true incidence may not be as low as previously believed.

**Electronic supplementary material:**

The online version of this article (doi:10.1186/s13000-015-0450-6) contains supplementary material, which is available to authorized users.

## Background

Malignant lymphomas (MLs) originate from aberrant monoclonal lymphocyte proliferation resulting from an interference with normal differentiation and ineffective control of growth. MLs usually involve a single histological type that remains unchanged during cancer development and progression, and the same type of ML can occur at different anatomic sites. A rarer condition known as composite lymphoma is defined by the presence of different types of MLs found at the same anatomic site [1], and an even rarer condition known as discordant lymphoma is defined by the presence of different ML types located at different anatomic sites [2]. The incidence of discordant lymphoma is believed to be very low, with fewer than 20 papers reporting cases of discordant lymphomas in the current literature. However, as we will discuss, some studies suggest the incidence many be higher than believed. A variety of non-Hodgkin lymphomas, Hodgkin lymphomas, and combinations of non-Hodgkin lymphoma and Hodgkin lymphoma have been observed in discordant lymphoma cases reported to date. Moreover, the coexistence of lymphoma and other malignant tumors also has been reported [3]. Among the combinations of non-Hodgkin lymphoma and Hodgkin lymphoma, several cases of B cell lymphomas and Hodgkin lymophoma combinations have been reported [4–6]. However, in most cases, the second tumor occurs after the treatment of the first one. It is rare that B cell lymphomas and Hodgkin lymophoma occur synchronously in discordant lymphoma. Here, we present a case of discordant lymphoma involving B cell lymphoma and Hodgkin lymphoma. To the best of our knowledge, this is the first reported case of simultaneous large B-cell lymphoma in the mediastinum and nodular sclerosis Hodgkin lymphoma in the right supraclavicular lymph nodes.

## Case Presentation

**Fig. 1:**
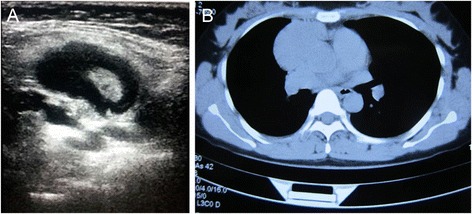
Representative images from ultrasound and CT examinations. **a** Ultrasound examination showed multiple hypoechoic nodules in the right supraclavicular lymph node. **b** CT scanning revealed an irregular soft tissue mass (6.2 cm × 3.7 cm) with a clear border in the mediastinum and suppression of the superior vena cava by the mass

A 34-year-old female patient was admitted to our hospital after a mass in the mediastinum was identified during a routine heath check-up. The patient reported no recent history of symptoms such as fever, chest pain, coughing, weight loss, or edema. The results of peripheral blood tests were all within the normal ranges. Multiple enlarged lymph nodes were identified at the right clavicle by palpation. Ultrasound examination showed multiple hypoechoic nodules (maximum size of 2.25 cm × 1.23 cm) on the right supraclavicular lymph nodes (Fig. 1a). Chest computed tomography (CT) examination showed an irregular soft tissue mass (6.2 cm × 3.7 cm) with a clear border in the mediastinum, and the superior vena cava was compressed by the mass (Fig. 1b). Pleural thickening and effusion were not observed. The mediastinal tumor and one of the affected right supraclavicular lymph nodes were surgically excised and pathologically examined.

Grossly, the mediastinal tumor was an encapsulated grayish-brown mass (7.5 cm × 6 cm × 4.5 cm) that did not exhibit bleeding or necrosis. The cut surface of the tumor revealed yellow and raw fish-like tissue. The tumors in the right supraclavicular lymph nodes were pale, bean-shaped nodules, and the cut surfaces of the tumors showed pale, solid tissue.

**Fig. 2:**
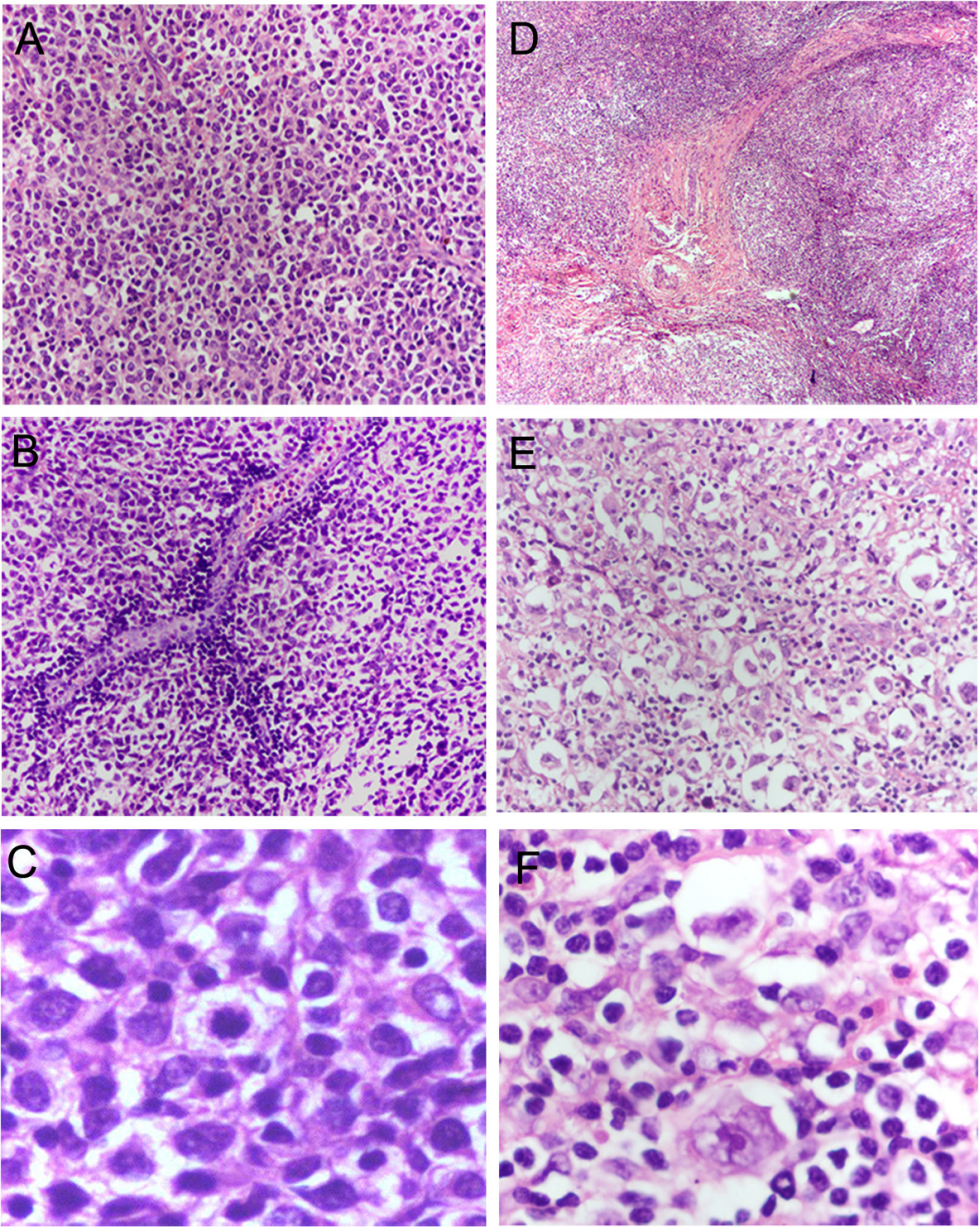
Histologic features of lymphomas observed via H&E staining. **a** Diffuse distribution of tumor cells (medium- or large-size) with lightly stained cytoplasm and heavily stained or bubbly nuclei was observed in the mediastinal mass (×200). **b** Large amounts of small lymphocytes formed a perivascular "angiolymphoid set" in the mediastinal tumor tissue (×200). **c** Mitosis was observed frequently in the mediastinal tumor cells (×400). **d** The right supraclavicular lymph node tissue was separated into nodules of varying size by refractive collagen fibers (×40). **e** Large tumor cells of varying size and distribution were scattered throughout the lymph node tissue (×200). **f** Lacunar cells were observed in lymph node sections (×400)

The excised tumors were characterized by histopathological examination. After fixation in 10 % neutral formaldehyde, the specimens were dehydrated in a graded series of ethanol solutions and embedded in paraffin for sectioning at 4 μm thickness. Histological examination via hematoxylin and eosin (HE) staining revealed that the encapsulated mediastinal tumor consisted of large nodules separated by thin fibrous tissues. Tumor cells (medium- or large-size) with lightly stained cytoplasm and heavily stained or bubbly nuclei were diffusely distributed throughout the tumor (Fig. 2a), and many small lymphocytes formed a perivascular "angiolymphoid set" in the tumor tissues (Fig. 2b). Small nucleoli and mitosis were observed frequently in the round or slightly irregular nuclei of tumor cells (Fig. 2c). In addition, residual thymic epithelial cells were observed in the encapsulated mediastinal tumor. Histological staining showed that the right supraclavicular lymph node tissue was separated into nodules of varying size by refractive collagen fibers and large tumor cells (mostly lacunar cells and a small amount of Reed-Sternberg cells, popcorn cells, and mummy cells) were scattered throughout the lymph node tissue (Fig. 2d–f). Some areas of necrosis were observed in the lymph node tissue, which was related to the infiltration of a large amount of inflammatory cells including small lymphocytes, eosinophils, plasma cells, and histiocytes.

**Fig. 3:**
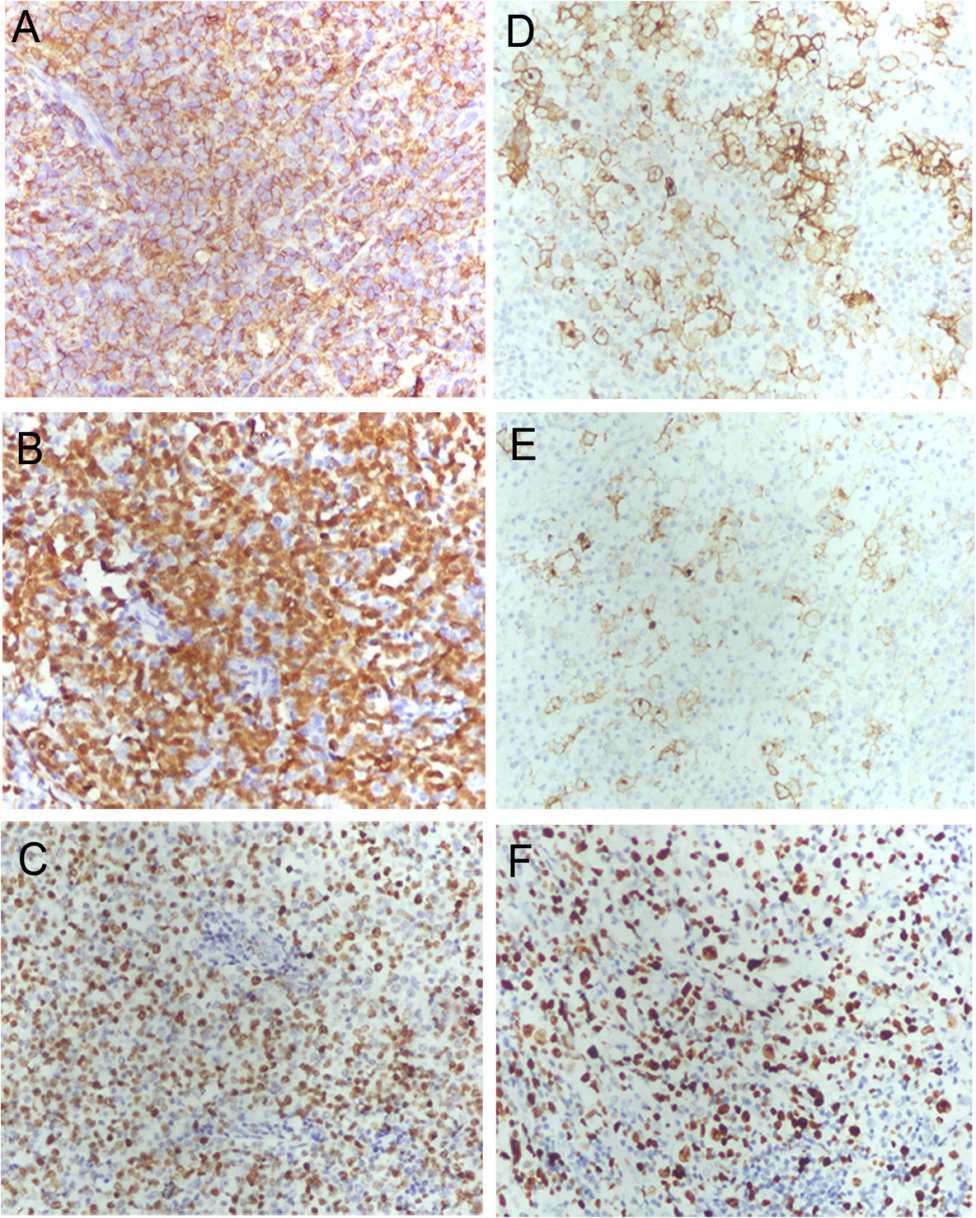
Expression of marker proteins for specific lymphoma types as determined by immunohistochemical staining. **a** CD79a in mediastinal tumor tissue (×200); **b** PAX5 in mediastinal tumor tissue (×200); **c** Ki67 in mediastinal tumor tissue (×200); **d** CD30 in the right supraclavicular lymph node tissue (×200); **e** CD15 in the right supraclavicular lymph node tissue (×200); **f** Ki67 in the right supraclavicular lymph node tissue (×200)

Tumor tissues also were examined using an immunohistochemistry (IHC) assay (EnVision™ two-step immunohistochemical technique) to determine the histological types of the tumors (Table 1). The antibodies (anti- PAX5, anti-CD20, anti-CD3, anti-CD5, anti-CD45, anti-CD79a, anti-CD10, anti- EMA, anti-CD57, anti-CD23, anti-CD30, anti- BCL-2, anti-CK, anti-CD21, anti-CD15, anti- MUM1, anti-BCL-6, and anti-Ki-67) used in the IHC assay were purchased from Zhongshan Golden Bridge, Inc. (Beijing, China). Cells within the encapsulated mediastinal tumor stained strongly positive (+++) for CD45 and CD79a (Fig. 3a), PAX5 (Fig. 3b), and CD20, and positive (+) for BCL-2 and MUM1. In addition, approximately 80% of the mediastinal tumor cells were positive (+) for Ki-67 (Fig. 3c). Small lymphocytes were positive (+) for CD3, and residual thymic epithelial cells were positive (+) for CK. In the right supraclavicular lymph node specimen, tumors cells stained strongly positive (+++) for CD30 (Fig. 3d), weakly positive (+) for CD15 (Fig. 3e), and negative (−) for EMA. Approximately 60% of tumor cells in the lymph node tissue were positive for Ki-67 (Fig. 3f). Small lymphocytes were strongly positive (+++) for CD3 and CD45, moderately positive (++) for CD79a and CD20, and weakly positively (+) for CD57, CD5, and CD23.

**Table 1:** Immunohistochemical staining results for the excised mediastinal mass and supraclavicular lymph nodes

	Mediastinal mass		Supraclavicular lymph node	
	Tumor cells	Small lymphocytes	Tumor cells	Small lymphocytes
CD3	-	+	-	+++
CD5	-	-	-	+
CD10	-	-	-	-
CD15	-	-	+	-
CD20	+++	-	-	++
CD21	-	-	-	-
CD23	-	-	-	+
CD30	-	-	+++	-
CD45	+++	-	-	+++
CD57	-	-	-	+
CD79a	+++	-	-	++
Bcl-2	+	-	-	-
Bcl-6	-	-	-	-
CK	-	-	-	-
EMA	-	-	-	-
MUM1	+	-	-	-
PAX5	+++	-	-	-
Ki67	80% +	-	60% +	-
EBER	-	-	-	-

Gene rearrangements and clonality analysis of immunoglobulin heavy chain gene, Kappa light chain gene, and Lambda light chain gene were identified in our case using the IdentiClone^TM^ IGH/IGK/IGL Gene Clonality Assay (InVivoScribe Technologies, CA, USA). The lacunar cells in nodular sclerosis Hodgkin lymphoma and the tumor cells in mediastinal large B-cell lymphoma micro-manipulated from tissues were subjected to single-cell PCR. Monoclonal gene rearrangement was observed in mediastinal large B cell lymphoma as two discrete bands appearing within 240–300 base pairs (bp) and 300–360 bp in the immunoglobulin heavy chain gene, but no monoclonal gene rearrangement was observed in nodular sclerosis Hodgkin lymphoma in the right supraclavicular lymph nodes (Additional file 1). Moreover, in our case, the EB virus-encoded small RNA (EBER) was used to design a single-stranded RNA probe for in situ hybridization (ISH) detection of Epstein-Barr (EB) virus (TIB Biosicences, Fuzhou, China). Nasopharyngeal cancer samples positive for EB virus RNA were used as a positive control in ISH experiments. Tissue samples from both the mediastinal tumor and a right supraclavicular lymph node were negative (−) for EB virus RNA.

Together these morphological, histopathological, and immunohistochemical observations as well as gene identification results, supported the diagnosis of discordant lymphoma consisting of mediastinal large B-cell lymphoma and nodular sclerosis Hodgkin lymphoma in the right supraclavicular lymph nodes. After surgical removal and evaluation of the mediastinal tumor and one right supraclavicular lymph node, iodine125 radiation particles were implanted in the remaining right supraclavicular lymph nodes, and the patient received six cycles of ABVD (doxorubicin [Adriamycin] + bleomycin + vinblastine + dacarbazine) chemotherapy. No metastasis or recurrence was observed during 32 months of follow-up examinations, suggesting that the treatment was successful.

## Discussion

As well as simultaneously presenting with other carcinomas [3], lymphomas can occur with other lymphomas at the same site or at different sites. The incidences of both composite lymphoma and discordant lymphoma are believed to very low. Composite lymphoma was first described by Custer in 1954 [1], and in the 60 years that have followed, the number of reported cases of composite lymphoma has steadily increased. However, discordant lymphoma cases have been reported in fewer than 20 papers to date, suggesting that the incidence of discordant lymphoma is lower than that of composite lymphoma. However, interestingly, Kim and Dorfman identified six (7 %) cases of discordant lymphomas among 84 untreated lymphoma patients [2]. In addition, Tucci et al. reported that 45 (13 %) of 347 lymphoma patients had multiple histological types of lymphomas, and 15 patients exhibited discordant lymphoma [7]. Considering the incidence of discordant lymphoma in these patient populations, it is possible that the overall incidence of discordant lymphoma may not be as low as previously believed. Based on the possible underestimation of the incidence of discordant lymphoma, clinical identification of one tumor may lead physicians to not continue searching for tumors at other sites. In some cases, discordant lymphoma patients have had no symptoms, such as the patient in the present case. The lack of symptoms can lead to missed diagnosis. These reasons may help to explain the low incidence of discordant lymphoma in the current literature.

As another consequence of the small number of cases reported in the literature, the pathogenesis of discordant lymphoma remains incompletely understood. The occurrence of discordant lymphoma may be an accidental phenomenon. Moreover, for cases of discordant lymphoma resulting from metachronous occurrence of different types of lymphomas, it seems plausible that a lymphoma at one site may have weakened the host immune system, which could increase the risk of another lymphoma occurring at another site [6]. In addition, EB virus has been shown to cause both Hodgkin lymphoma and non-Hodgkin lymphoma in the same patients [8], and both Hodgkin lymphoma and non-Hodgkin lymphoma at different sites were observed in HIV-positive patients [9]. However, no EB virus RNA was detected in the tumor tissues in the present case, indicating that EB virus was not involved in the development of the lymphomas in this case. Furthermore, it has been reported that anti-tumor therapy against a first lymphoma increases the incidence of a second lymphoma due to the toxic effects of anti-tumor treatments, which can lead to discordant lymphoma [5, 8]. However, Van den Tweel et al. presented an inconsistent view with the suggestion that discordant lymphomas originate from the same lymphocytes but exhibit different morphologies at different stages [10].

Although few in number, the previous reports of discordant lymphoma cases illustrate that the histological types of tumors present in cases of discordant lymphomas can be very diverse. Examples of previously reported discordant lymphoma cases are listed in Table 2. Hodgkin lymphomas and non-Hodgkin lymphomas have been the most commonly reported histological types in discordant lymphomas [4, 11, 12]. Among these combinations, several cases of B cell lymphomas and Hodgkin lymophoma combinations were reported previously. In addition, one study revealed that a B-cell lymphoma may be present at one site and a T-cell lymphoma at another site [13], while other studies demonstrated that different B-cell lymphomas may be present at multiple sites [14, 15]. Moreover, a variety of lymphoma types can occur either synchronously or metachronously at different sites. But, it is difficult though to determine clinically whether lymphomas at different sites occur synchronously or metachronously. The patient in our case was sought treatment without any related symptoms or medical history. Therefore, we speculate that the two lymphoma types occurred synchronously.

**Table 2:** Cases of discordant lymphoma reported from 2000 to 2015 and the current case

no./Sex/Age	Lacation	Diagnosis	Therapy	Outcome	Ref.
1/F/52	Inguinal LN	HL	ABVD	CR	5
	Nasopharyngeal	B cell NHL	NA	NA	
2/M/44	Intraabdominal mass	FL	chemotherapy	CR	6
	Cervical LN	HL	NA	NA	
3/F/67	Submandibular LN	HL	ABVD	CR	8
	Skin	PTCL	CHOP	Died	
4/M/32	Postcervical LN	HL	ABVD	CR	11
	Submandibular LN	PTCL	Autologous PBSCT	NA	
5/M/60	Inguinal LN	PTCL	Autologous PBSCT	NA	11
	Cervical LN	HL	CHOP	Recurrence	
6/M/40	Inguinal LN	HL	EVA	CR	11
	Cervical LN	HL	EVA	CR	
	Subcarinal LN	ALCL	NA	Died	
7/F/77	Cervical LN	PTCL	CHOP	Recurrence	11
	Skin	Necrotic T-cell NHL	NA	NA	
8/M/37	Cervical LN	PTCL	IVAM, BEAM Autologous PBSCT	Recurrence	12
	Supraclavicular,	HL	ABVD	Died	
	Inguinal, et al.				
9/M/55	Cervical LN	DLBCL	Chemotherapy	Died	13
	Bone marrow	PTCL	CHOP	Died	
10/F/50	Stomach	MALT	NA	NA	14
	Parotied gland	FL	NA	NA	
11/M/70	Skin	FL	NA	NA	15
	LN	MCL	NA	NA	
12/F/34	Supraclavicular LN	HL	Iodine 125 radiation	CR	present
	Mediastinum	DLBCL	ABVD	CR	study

*LN* lymph node; *CR* complete remission; *NA* not available; *PTCL* peripheral T-cell lymphoma; *HL* Hodgkin lymphoma; *ALCL* anaplastic large cell lymphoma; *NHL* non-Hodgkin lymphoma; *MALT* mucosa-associated lymphoid tissue; *FL* follicular lymphoma; *MCL* mantle cell lymphoma; *MZL* marginal zone lymphoma; *PBSCT* peripheral blood stem cell transplant; *ABVD* doxorubicin (Adriamycin), bleomycin, vinblastine, dacarbazine; *CHOP* cyclophosphamide, doxorubicin, vincristine (Oncovin), prednisone; *EVA* etoposide, vinblastine, Adriamycin; *IVAM* ifosfamide, etoposide, cytarabine, methotrexate; *BEAM* bendamustine, etoposide, cytarabine, melphalan

We now have confirmation that Hodgkin lymphoma originates from B cells. Previous reports indicated the possibility of transformation between B cell lymphomas and Hodgkin lymphomas, including that large B cell lymphomas can transform into Hodgkin lymphomas or Hodgkin lymphomas can transform into large B cell lymphomas [16]. In most cases of discordant lymphoma with B cell lymphomas and Hodgkin lymophoma combinations, the second tumor occurs after the treatment of the first one. It is suspected that the transformation between these two lymphomas was induced by the treatment and both tumors may have originated from the same cell line. This speculation was confirmed in the study by Nakamura et al. [6], who performed polymerase chain reaction (PCR) amplification of the bcl-2 and IgH genes and then demonstrated the common clonal origin of follicular lymphoma and subsequent Hodgkin lymphomas. Large B-cell lymphoma and Hodgkin lymphoma originate from the same cell type (B cells). However, these two tumors in the spectrum of B-cell lymphomas have completely different morphological features and immunophenotypes, and they belong to two separate neoplasms. Thus, they are clearly distinguished and named in the WHO classification of lymphoid tissues [17]. However, Rosenwald et al. [18] used gene expression profiling to suggest over one third of the genes that were more highly expressed in PMBL than in other DLBCLs were also characteristically expressed in Hodgkin lymphoma cells. In this case, patient had no obvious symptoms or the history of anti-tumor treatment. One tumor in the mediastinum presented the typical morphology and phenotypic features of large B-cell lymphoma, and the other one in the right supraclavicular lymph nodes presented the typical morphology and phenotypic features of nodular sclerosis Hodgkin lymphoma, consistent with the obervastion that EBER-1 RNA ISH failed to stain in both lymphomas. Furthermore, we determined the gene rearrangements and performed a clonality analysis of the immunoglobulin heavy chain gene, Kappa light chain gene, and Lambda light chain gene and demonstrated monoclonal gene rearrangement in the immunoglobulin heavy chain gene in mediastinal large B-cell lymphoma. In contrast, no monoclonal gene rearrangement was observed in nodular sclerosis Hodgkin lymphoma in the right supraclavicular lymph nodes. These findings suggest that these two lymphomas were not of a common clonal origin. Consequently, the diagnosis was definitive for both tumors according to medical history, histopathological, immunohistochemical observations, and gene identification.

Cases of discordant lymphoma are more complicated and difficult to treat than those involving a single tumor. Thus, the prognosis of discordant lymphoma is generally worse than that for cases of a single lymphoma. Determining the best therapeutic strategy for treating multiple lymphomas simultaneously is difficult, and the treatment strategy typically is commonly based on the more malignant lymphoma type. Fortunately, some target drugs gene therapy have been reported [19], such as JAK2 selective inhibitor, basing on disease-specific chromosome 9p24.1/JAK2 amplification increased JAK2 expression and activity in bothprimary mediastinal large B-cell lymphoma and Hodgkin lymphoma. Therefore, early diagnosis and accurate determination of the histological types of all tumors are critically important for individualized treatment and favorable prognosis of discordant lymphoma.

## Conclusion

The diagnosis of discordant lymphoma, subsequent differentiation of the involved lymphomas, and eventual treatment planning are challenging due to the complex, highly variable, and poorly understood pathogenesis of this condition. Additional research is needed to determine an accurate incidence of discordant lymphoma, given that current underestimation of this condition may lead to missed diagnosis and thus worsen patient outcomes.

## Consent

Written informed consent was obtained from the patient for publication of this case report and any accompanying images. A copy of the written consent is available for review by the Editor-in-Chief of this journal.

## Additional file

Additional file 1:
**Gene rearrangements and clonality analysis of immunoglobulin heavy chain gene, Kappa light chain gene, and Lambda light chain gene were identified in mediastinal large B-cell lymphoma and in nodular sclerosis Hodgkin lymphoma in the right supraclavicular lymph nodes using the IdentiCloneTM IGH/IGK/IGL Gene Clonality Assay (InVivoScribe Technologies, CA, USA).** (TIF 1129 kb)
