# Supplementary figures and images for: Discordant lymphoma consisting of mediastinal large B-cell lymphoma and nodular sclerosis Hodgkin lymphoma in the right supraclavicular lymph nodes: a case report
Source: Diagn Pathol. 2015 Dec 29;10:215. doi: 10.1186/s13000-015-0450-6 (PMC4693434; doi:10.1186/s13000-015-0450-6)

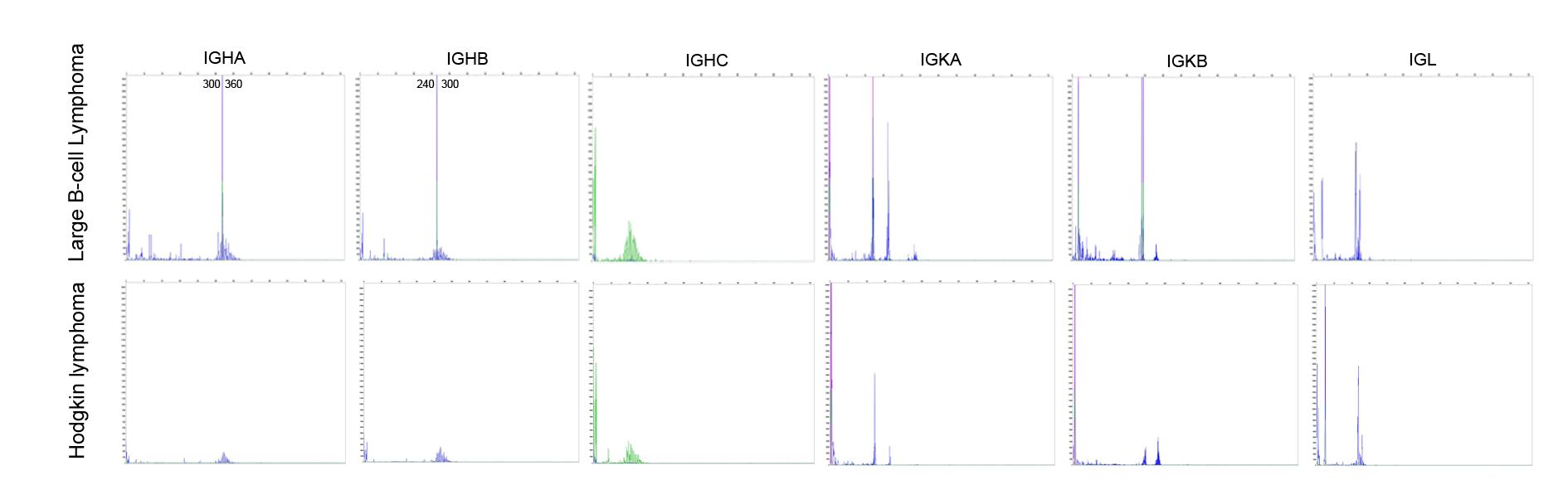

Supplement: Additional file 1: — Gene rearrangements and clonality analysis of immunoglobulin heavy chain gene, Kappa light chain gene, and Lambda light chain gene were identified in mediastinal large B-cell lymphoma and in nodular sclerosis Hodgkin lymphoma in the right supraclavicular lymph nodes using the IdentiCloneTM IGH/IGK/IGL Gene Clonality Assay (InVivoScribe Technologies, CA, USA). (TIF 1129 kb) [file 13000_2015_450_MOESM1_ESM.tif]
